# The Detection of Novelty Relies on Dopaminergic Signaling: Evidence from Apomorphine's Impact on the Novelty N2

**DOI:** 10.1371/journal.pone.0066469

**Published:** 2013-06-20

**Authors:** Mauricio Rangel-Gomez, Clayton Hickey, Therese van Amelsvoort, Pierre Bet, Martijn Meeter

**Affiliations:** 1 Department of Cognitive Psychology, VU University Amsterdam, The Netherlands; 2 Department of Psychiatry and Psychology, Maastricht University, Maastricht, The Netherlands; 3 Department of Clinical Pharmacology and Pharmacy, VU University Medical Center, Amsterdam, The Netherlands; University G. D'Annunzio, Italy

## Abstract

Despite much research, it remains unclear if dopamine is directly involved in novelty detection or plays a role in orchestrating the subsequent cognitive response. This ambiguity stems in part from a reliance on experimental designs where novelty is manipulated and dopaminergic activity is subsequently observed. Here we adopt the alternative approach: we manipulate dopamine activity using apomorphine (D1/D2 agonist) and measure the change in neurological indices of novelty processing. In separate drug and placebo sessions, participants completed a von Restorff task. Apomorphine speeded and potentiated the novelty-elicited N2, an Event-Related Potential (ERP) component thought to index early aspects of novelty detection, and caused novel-font words to be better recalled. Apomorphine also decreased the amplitude of the novelty-P3a. An increase in D1/D2 receptor activation thus appears to potentiate neural sensitivity to novel stimuli, causing this content to be better encoded.

## Introduction

The ability to respond accurately and rapidly to novel stimuli relies on a cascade of neurological mechanisms that underlie perception, attention, learning and memory [1]. Although stimulus novelty has received much study, it is still not certain how novelty detection occurs, which structures are involved, and what neurotransmitter systems intervene.

Event-Related Potential (ERP) markers are ideally suited to understand the neuromodulatory mechanisms of novelty processing. Novel stimuli usually elicit two ERP components in succession: the anterior novelty N2 (N2b in Pritchard and colleagues [2] division of the N2), and the P3, associated with the allocation of attention to the novel stimulus [3], [4]. The N2 generally appears to reflect processing involved in the automatic detection and recognition of novel stimuli [5], [6], and the component is greatly reduced after a single repetition of a novel stimulus [7]. It has been decomposed into three subcomponents: the N2a, N2b and N2c [2]. These correspond to the mismatch negativity (N2a), the anterior N2 or novelty N2 (N2b) and posterior N2 (N2c; [8]). The N2a/mismatch negativity has a fronto-central maximum distribution and is thought to reflect an automatic neural response to an auditory outlier [9], [10], whereas the N2b commonly precedes the P3a component and is commonly elicited in the visual oddball task [11], [12]. The latter component is considered semiautomatic, in that it is elicited by oddball stimuli regardless of task relevance [5], [6]. The N2c, which commonly precedes the P3b component, is associated with classification tasks [13].

The P3 component has also been divided in two subcomponents: the fronto-central P3a (or novelty P3) and the centro-parietal P3b. The P3a has been associated with the evaluation of novel stimuli for subsequent behavioral action and is postulated to be a marker of a conscious attentional switching mechanism [14] and possibly an index of distractibility [15]. The P3b is rather though to index processes related to the recognition of stimulus meaning and significance [4], [7]. Consistent with this, the P3b is enhanced for stimuli that are related to later decisions or responses [16].

Several pharmacological studies have employed the N2 and P3 to explore the molecular basis of novelty detection, mostly with drugs that affect a broad range of neurotransmitters. Soltani and Knight [17], in a comprehensive literature review, suggest that the amplitude of the oddball-elicited P3 is dependent on the operation of several monoamines, particularly dopamine and norepinephrine. Consistent with this, Gabbay and colleagues [18] found that d-amphetamine, a non-selective dopamine and norepinephrine agonist, alters P3a, N100 and reorienting negativity (RON) reactivity to novel stimuli. Participants with a preference for d-amphetamine presented larger P3a amplitude, reduced amplitude N100 and reduced amplitude RON after d-Amphetamine, as compared to participants with no preference for the drug.

More specific pharmacological interventions have been used in research with animals or in studies in which patients are tested in conditions on and off medication. In schizophrenia, which is associated with dysfunctions in the dopamine system, the mismatch negativity (MMN) is reduced when patients receive neuroleptic treatment that blocks dopaminergic pathways [19]. In a study with Parkinson’s disease (PD) patients the administration of L-Dopa or dopaminergic agonists did not change novelty preferences, as assessed by a three-armed bandit task. However, this finding is difficult to interpret due to comorbidity in the sample, which included patients with impulsive compulsive behaviors [20].

Other studies have employed a correlational approach, in which activation in certain regions and neurotransmitter gene polymorphisms have been associated to indexes of novelty processing. Functional Magnetic Resonance Imaging (fMRI) data shows novelty-elicited activity in dopamine-rich mesolimbic areas like the substantia nigra and ventral tegmental area [21]. Polymorphisms of genes related to dopamine availability (COMT) and density of D2 receptors (ANKK1) have been found to modulate the processing of novelty, such that higher P3a amplitude is related with the balance of these two variables [22]. Genes encoding for dopaminergic transporters (DAT1) have also been implied in the detection of task novelty [23]. These studies suggest that higher dopaminergic availability enhances the detection and further processing of novel stimuli. Additionally, P3a amplitude is reduced when dopamine levels are low, as shown by studies with Parkinson’s disease patients [24], [25].

However, in a recent review Kenemans and Kähkönen [26] suggest that the effect of dopamine manipulation on novelty related components, like the MMN and the P3, is weak, and that the main effect of dopamine is rather on subcortical processing related to conflict monitoring. These authors also suggest that the effect of dopamine is receptor dependent, and that agonism of the D1/D2 receptors is implicated in the speeding of perceptual processes.

Though the evidence discussed above suggests a function for dopamine in novelty processing, the precise nature of this role remains unclear. It may be that dopamine acts to create neural *sensitivity* to novel stimuli, thus playing a critical part in novelty detection [27]. Alternatively, novelty-induced activity in dopaminergic brain areas may reflect a subsequent *reaction* to novel stimuli, indexing the cognitive response to environmental events that are likely to be behaviorally relevant [28].

In the present study we manipulated the dopamine system by the administration of the D1/D2 agonist apomorphine and measured novelty-related ERP components. This approach allows us to disentangle the role of dopamine in novelty processing [29], [30]. Participants completed two experimental sessions, one following administration of apomorphine and one following administration of a saline placebo. In order to determine D1/D2 receptor involvement in novelty processing we had participants complete a von Restorff task in each session while electroencephalogram was recorded. In this task, participants study a list of words, some of which stand out because of unique font and color. These are subsequently better remembered [31] because of their relative novelty [32].

Extant ERP studies of novelty processing have tended to employ 'oddball' paradigms rather than the von Restorff task. In the standard oddball task the physiological response to infrequent non-standard stimuli is assessed. This task requires participants to respond to a specific target that is presented in a sequence of stimuli that also contains infrequent, task-irrelevant novel stimuli. We used the less-common von Restorff task for two reasons. First, it provides a behavioural index of novelty processing, namely recall rates for novel stimuli. Second, novelty-induced changes in recall constitute a measure of the impact of novelty on memory and learning. As noted above, the current study was motivated by the idea that dopamine may impact learning through its role in novelty detection, and our fundamental interest is in how novelty comes to impact learning and memory. We thus chose employ a task that allows perspective on how novelty impacts these subsequent cognitive processes (see also [33], [34].).

If dopamine D1/D2 receptor activation increases the sensitivity of the brain to novelty, our expectation was that the stimulation of dopamine receptors caused by apomorphine would create a larger novelty N2 to novel font words. If dopamine is rather involved in the subsequent cognitive reaction, this should be reflected in later components like the P3a, but the N2 should be unaffected.

## Results

### Behavioral Data

**Figure 1:**
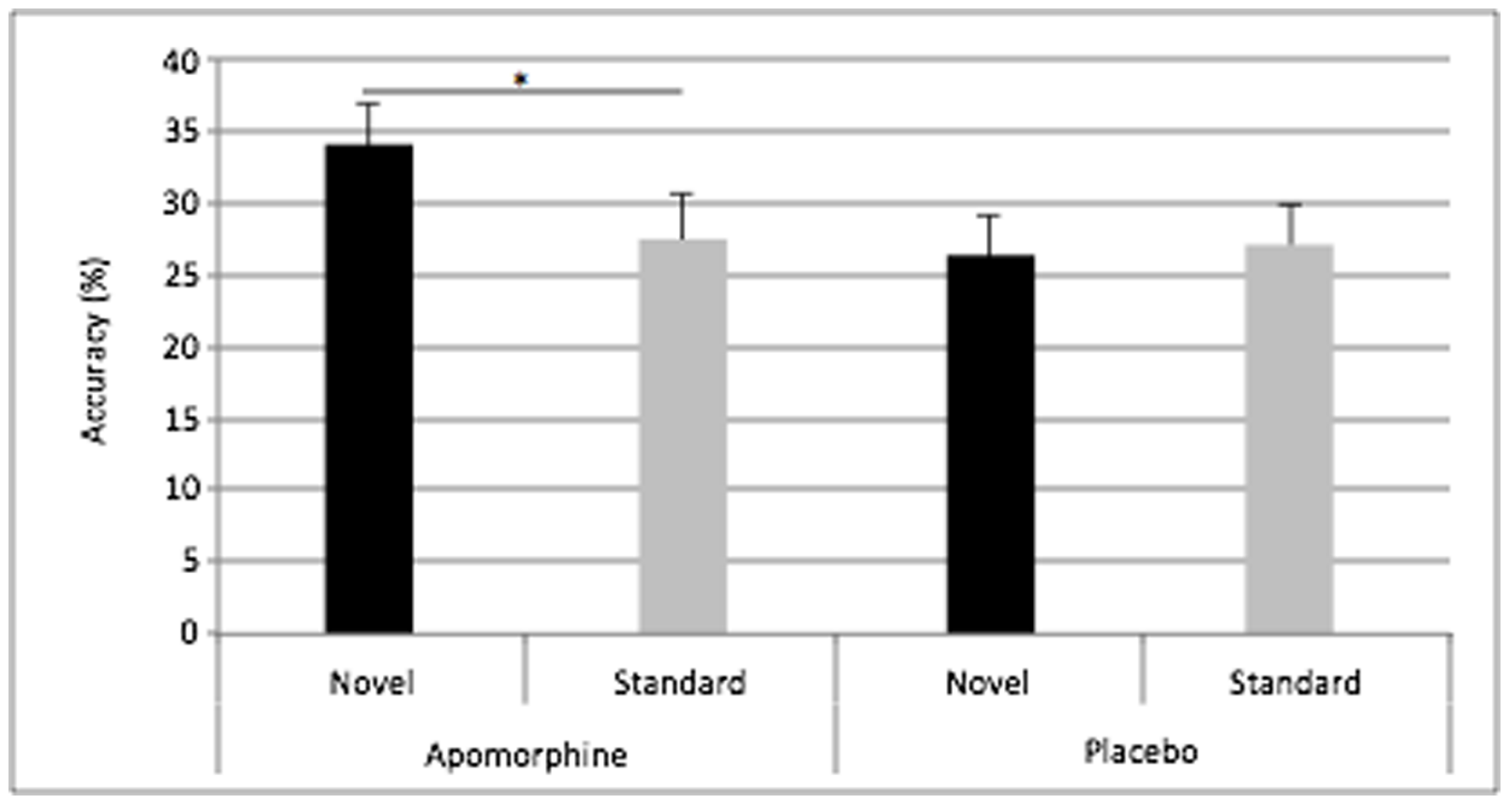
Behavioral data. Accuracy (%) is presented in the y-axis, and bars are plotted for novel and standard stimuli, both under apomorphine and placebo. Error bars represent the standard error of the mean of the individual means.

Figure 1 presents recall accuracy as a function of font novelty (novel/standard) and drug condition (apomorphine/placebo). Mean accuracy across drug conditions for novel words was 30.2% and for standard words 27.3%. Statistical analysis took the form of a repeated measures analysis of variance (RM ANOVA) with factors for novelty and drug condition. This revealed no main effect of drug condition (*F*_1,25_ = 2.27, p = 0.143), no main effect of novelty (*F*_1,25_ = 2.02, P = 0.174), but, critically, an interaction between the factors (*F*_1,25_ = 4.32, p = 0.048). Follow-up contrasts demonstrated that performance for novel font words was better than that for standard font words in the apomorphine condition (*t*_25_ = 2.61, p = 0.015), but that there was no recall difference between novel font and standard words in the placebo condition (*t*_25_ = 0.12, P = 0.913). Note that statistical values for these planned contrasts reflect raw, uncorrected values.

### ERP Data

**Figure 2:**
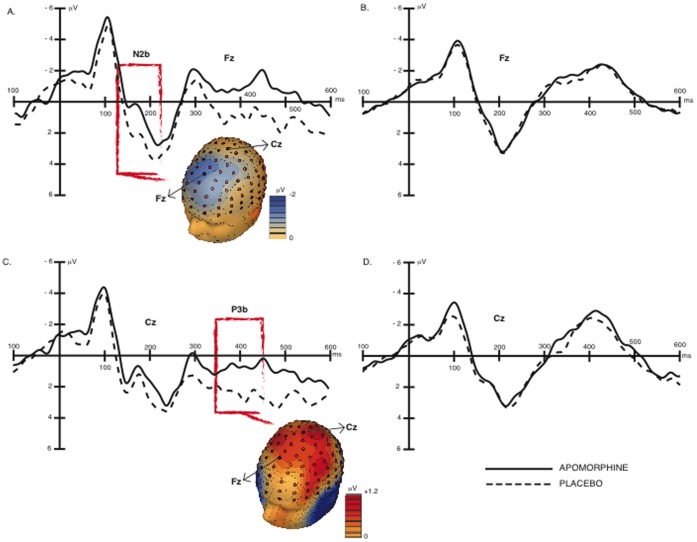
ERPs elicited by novel and standard stimuli after administration of apomorphine and placebo, at electrodes Fz and Cz. Time is indexed on the x-axis in milliseconds; zero indicates stimulus onset. ERP magnitude is indexed on the y-axis in microvolts (µV). For all the ERP plots, the continuous line corresponds to the Apomorphine condition and the dotted line to placebo. A. ERPs to novel stimuli at Fz electrode. Additionally the topography of the difference between apomorphine and placebo conditions is presented, as calculated from 140 ms to 220 ms. B. ERPs to standard stimuli at Fz electrode. C. ERPs to novel stimuli at Cz electrode. Additionally the topography of the difference between apomorphine and placebo conditions is presented, as calculated from 350 ms to 450 ms. D. ERPs to standard stimuli at Cz electrode.

As standard font words did not elicit a clear N2 (see right panel of figure 2) we identified the N2 component based on the response to novel font stimuli (see left panel of figure 2). Consistent with existing literature [6], the N2 was maximal at fronto-central electrode sites roughly corresponding to Fz and FCz in the 10–10 electrode naming convention. The plots presented in Figure 2 reflect the potentials recorded at midline electrodes approximately equivalent to the electrodes Fz and Cz of the 10–10 electrodes system.

As illustrated in the top-left panel of Figure 2, the N2 observed in the apomorphine condition was both earlier and larger when elicited by novel font words. The N2 overlaps with a concurrent positive component, the P2, which itself peaks around 180 ms. However, the topographical distribution and the observed latency differences between drug and placebo conditions point to a specific modulation of the N2.

We began statistical analysis by testing the reliability of the N2 latency shift. This was achieved through the use of a jackknifed bootstrap procedure in which N2 onset latency was defined as the moment when this component reached 50% of its maximum amplitude (see [35].) This analysis demonstrated that the N2 onset is earlier in the apomorphine condition (166 ms) than in the placebo condition (176 ms; *t*_25_ = 2.19, p = 0.041).

Given this pattern our analysis of N2 amplitude is based on different latency intervals for the apomorphine and placebo conditions. For each condition, we calculated mean amplitude observed across a 20 ms interval centered on the peak of the N2 [36]. Thus, the N2 for the apomorphine condition was defined as mean amplitude between 156 and 176 ms, and for the placebo condition between 166 and 186 ms. Results showed a reliably larger N2 in response to novel font stimuli in the apomorphine than in the placebo condition (*t*_25_ = 2.88, p = 0.008). See Table 1.

**Table 1:** Description of a control experiment, designed to test the effect of size on the expression of the ERP components presented in this document.

Novel font words were not only presented in a unique font and color but also in a larger font size, raising the possibility that the novelty related differences reflect only size differences. To test this possibility we performed a control experiment where we reversed the size relationship, presenting standard font words in large 30 pt font and novel words in small 17 pt font. As in the current study, in the control study the anterior N2 and P3a were numerically larger to novel-font words, not to the physically larger standard-font stimuli, though not significantly so.

We observed no drug-induced differences in amplitude of the P3a (∼250–350 ms. post-stimulus). In contrast, the P3b elicited by novel font words appears smaller in the apomorphine condition (top-left panel of Figure 2). Peak P3b amplitude was observed at posterior electrode sites, and statistical analysis was accordingly based on mean potential observed from 350–450 ms post-stimulus at an electrode located on a position corresponding to the Cz label in the 10–10 montage. This analysis revealed a reliable decrease in the P3b amplitude elicited by novel font words in the apomorphine condition as compared to the placebo condition (*t*_25_ = 2.37, p = 0.026).

## Discussion

We investigated the role of dopamine D1/D2 receptor activation in the processing of novel stimuli. Following administration of the D1/D2 agonist apomorphine we had participants perform a memory task involving the presentation of novel-font word stimuli. EEG was recorded while participants completed the task and we isolated the novelty-induced anterior N2 and P3a ERP components.

Given that the anterior N2 has been related to the detection of stimulus novelty [5], [6], and is thought to index action of a novelty detection network located largely in frontal cortex [37], [38], it can be used as an index of novelty detection within the context of a pharmacological intervention that affects the dopamine system. Existing work suggests that dopamine is involved in the detection of novelty [23], and specifically with the speed of perceptual processes [26]. If D1/D2 receptor activation plays a critical role in the detection of novelty, our expectation was that apomorphine should have a marked impact on the anterior N2. Consistent with this, this component was reliably larger and earlier in the apomorphine condition.

Importantly, the impact of apomorphine on the anterior N2 identified in our study contrasts with effects of apomorphine observed in earlier work. For example, in Ruzicka et al. [29] administration of apomorphine to Parkinson's patients caused the N2 and P3 elicited by auditory target stimuli to be smaller and later than those elicited in off-drug conditions. Ruzicka et al. concluded that apomorphine slows cognitive processes underlying discrimination and categorization (see also [29], [39], [40]), much as is observed following administration of levodopa in Parkinson's patients (e.g. [41)]. In this context the speeding and amplification of the N2 apparent in our results is striking: apomorphine appears to have an effect specifically on the novelty-induced N2 that is directly opposite to the general slowing seen in N2 and P3 components in earlier studies.

Consistent with this prior work demonstrating a generally disruptive effect of apomorphine, we found a broad reduction in P3 amplitude - particularly in the P3b - when participants were under the influence of the drug (see Figure 2). These results are inconsistent with previous genetic evidence, which relate enhanced dopaminergic activity with increased amplitude of P3a [42]. On the face of it, this might suggest a negative impact of the drug on the attentive and mnemonic mechanisms indexed by the P3. However, consistent with other findings in the literature [40], our results showed no relationship between catecholomine-induced P3 variability and behavioural performance. Apomorphine in fact had a reliably *beneficial* impact in the recollection of novel-font words.

This pattern suggests to us that variation in recall of novel-font words - the von Restoff effect - is reflected in the anterior N2, not the P3, and thus reflects change in the neural sensitivity to novelty rather than subsequent cognitive processes. This is consistent with a body of findings from our lab showing a dissociation between P3 amplitude and the probability that a novel-font word will be recalled [43]. The apparent absence of any drug effect on the P3a could alternatively reflect the combined impact of two concurrent drug influences: on the one hand apomorphine may act to increase P3a amplitude by enhancing sensitivity to novelty (as suggested by the current N2 results), on the other hand apomorphine may act to decrease P3a amplitude through its broader negative impact on the amplitude of ERP components.

As noted above, existing work suggests that apomorphine has a generally disruptive impact on cognition, but our results clearly demonstrate that it facilitates the novelty detection mechanisms indexed by the anterior N2. This is in line with the ideas of Redgrave and Gurney [27], who argue that novel, unexpected stimuli cause fast, automatic dopamine release. The role of this release would be to sensitize other brain areas to the occurrence of novel environmental configuration, and facilitate the learning both of these stimuli and of the responses that may have caused their appearance. Novelty in this way becomes key to behavioral plasticity - setting the stage, through dopamine, for learning.

As evident in Figure 2, the N2 component observed in this study overlaps with the P2 component of the ERP, and our results may accordingly reflect a combination of effects on these two components. Both the N2 and P2 occur in much the same latency interval and are difficult to distinguish (other than by polarity) as they are largely sensitive to the same experimental manipulations and have much the same topography. They appear to reflect activity in physically close generators, if not in the same brain structures (as would be possible if the polarity difference were due to cortical folding).

However, it is very unlikely that variations in the P2 can solely account for our results. First, P2 amplitude elicited by standard fonts was not influenced by apomorphine, consistent with existing results suggesting that the P2 is susceptible to task relevance rather than novelty [44]. Second, it is unlikely that the observed N2 latency shift could be created by change in the P2. The N2 is a relatively high-frequency component in this dataset, whereas the P2 is of lower frequency (and comes to sum with P3a). Variation in this low-frequency positive-polarity complex is unlikely to create a shift in the higher-frequency N2 peak.

We propose that the current results reflect variation in the anterior N2, but an alternative interpretation might be that our experimental manipulation impacts the mismatch negativity [45], [46]. However, previous studies suggest that dopamine has no influence in the generation or modulation of the MMN [47]. Moreover, the generators of the visual MMN appear to be located in posterior cortex, with a maximum over occipital areas [48] rather than at the anterior locations evident in our results.

We therefore conclude that apomorphine has an impact on novelty processing as indexed in the anterior N2. Apomorphine is generally thought to have an agonistic impact on D1/D2 receptors, consistent with the idea that increased activity in the dopamine system can be associated with increased sensitivty for novel stimuli. However, two caveats must be attached to this idea. First, it remains unclear whether apomorphine at low dosage acts as an agonist, or rather as an effective antagonist through its impact on autoreceptors [49], [50]. This potential antagonistic effect has been suggested as an explanation for detrimental cognitive effects in Parkinsonian patients [51], [52], but has yet to be conclusively demonstrated. In our study, apomorphine had no effect on baseline memory, but selectively improved memory for novel stimuli. The idea that a dopamine antagonist would create this pattern is hard to reconcile with any current theoretical account. In contrast, if that apomorphine acted as an agonist, this behavioural improvement is highly consistent with the idea that dopamine is involved in novelty detection.

Second, our interpretation is based on the idea that the central experimental manipulation is stimulus novelty. Novel font words also differed from standard font words in the physical features of color, size and font type, which could theoretically also play a role in the generation of the responses analyzed here. However, it is not likely that these physical features would elicit responses such as the N2 and P3a, and this was controlled for in the case of size in a control experiment. Moreover, variation in these types of stimulus feature show no correlation with changes in activity of dopamine-rich midbrain nuclei [53].

In conclusion, our results show that administration of the D1/D2 agonist apomorphine led to enhanced detection of stimuli with novel color, font, and size, as reflected in earlier onset and increased amplitude of the anterior N2 component of the ERP. To our knowledge, this is the first study to show that activation of D1/D2 receptors selectively increases the brain’s sensitivity to novelty. The role of this increased sensitivity could be to facilitate learning of novel stimulus configurations and the responses associated with them. Consistent with this, we found that novel objects are better recalled after D1/D2 receptor activation.

## Experimental Procedure

### Participants

Twenty-six healthy volunteers with normal or corrected-to-normal vision were recruited from the student population of the VU University Amsterdam. None of the participants reported any known neurological or psychiatric pathology. All participants gave written informed consent and received €150 for participation in the study plus compensation for travel costs. The participants group was composed of 17 females and 9 males, with ages ranging from 18 to 32 years (mean, 22 yr; s.d., 3.9 yr). Twenty-three of the participants were right handed. The study was performed in agreement with the Declaration of Helsinki and approved by the ethics committee of the VU University Amsterdam.

### Pharmacological Intervention

Participants were tested once after subcutaneous administration of apomorphine and once after placebo, double-blinded. The two testing sessions were scheduled one week apart to reduce carry-over effects, and order of sessions was counter-balanced across participants.

In the apomorphine session the drug was administered by a certified researcher at a ratio of 0.005 mg/kg. Apomorphine was obtained from Brittannia Pharmaceuticals Ltd. (commercial name Apo-Go). In the placebo session saline was administered in the same manner and volume. Doses of apomorphine and saline were delivered to the researcher in indistinguishable injection needles with coding retained by the pharmacy.

Thirty minutes before administration of both apomorphine or placebo participants received a 40 mg oral dose of domperidone, a D2 antagonist that selectively impacts the peripheral nervous system (See also [52]). Domperidone was obtained in oral tablets of 10 mg from Johnson & Johnson (commercial name Motilium), and was administered to counteract known side effects of D2 agonists, which include nausea and somnolence [54]. Nevertheless, 11 participants reported nausea and somnolence after administration of apomorphine. Consistent with existing work employing this combination of drugs [52], [55], these side effects were short-lived, generally lasting no more than 15 minutes, and participants reported being alert and task-ready after this interval.

### Procedure and Stimuli

**Figure 3:**
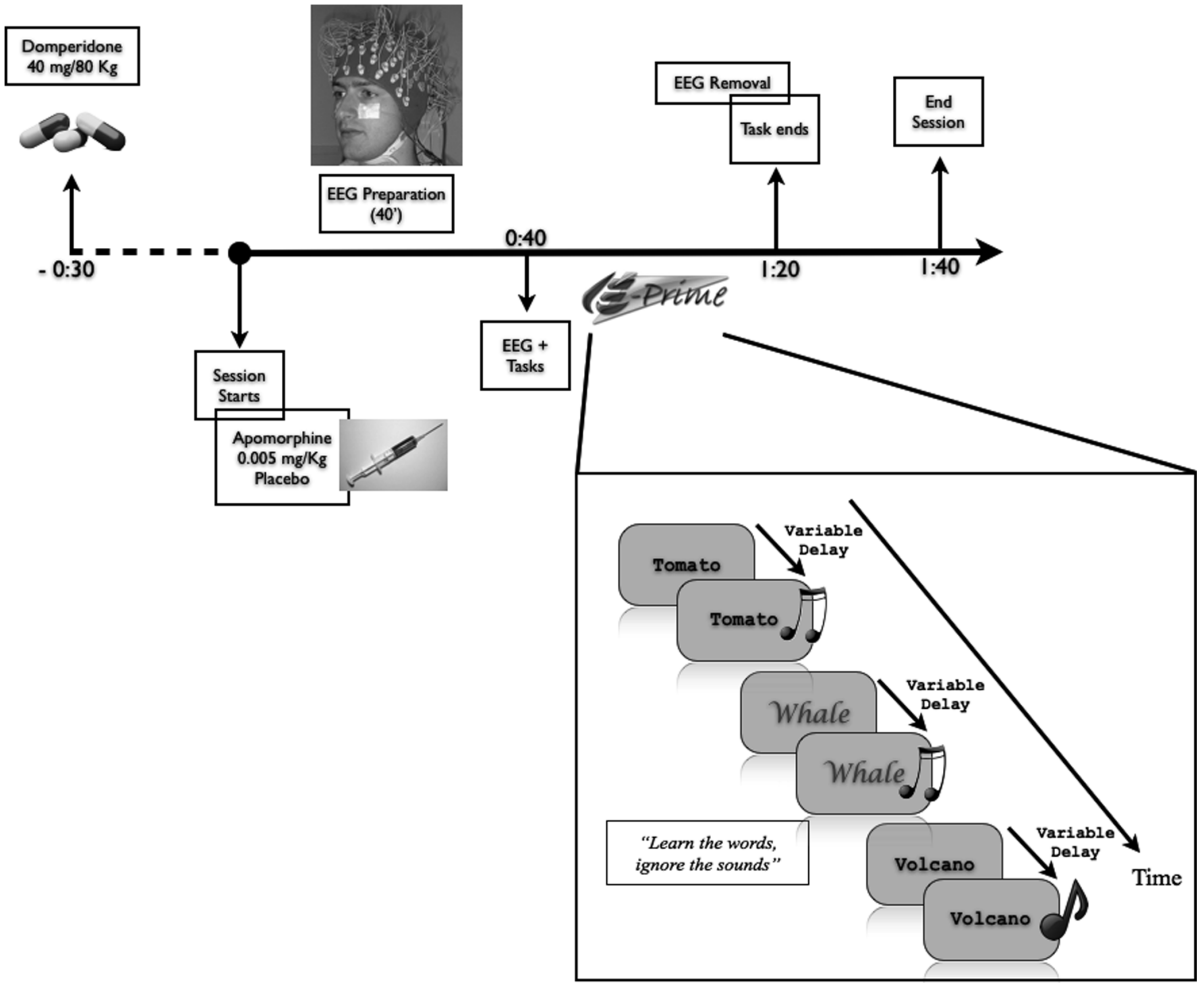
Schematic representation of the testing session and task (cutout). Words were presented once every 3.5 seconds. Some words, shown here in white, had a unique font and color (novel font). During the presentation of each word, a sound was played with a variable delay after word presentation. For most words this was a standard sound, for some a unique sound (novel sound, shown here with Volcano).

Figure 3 shows a schematic representation of the testing session. As apomorphine has a 40 to 50 minute rise time, so testing started forty minutes after injection [52], [55]. We employed a modified von Restorff verbal learning task in which words presented in standard font and words presented in novel font are studied and later recalled. Novel font words are typically remembered better than standard font words [31]. A schematic representation of the task is shown in Figure 3. It consisted of a study phase, cued recall phase, and final recognition phase, but performance during the final recognition phase was at ceiling and only results from the cued recall phase are discussed below.

During the study phase, participants were presented with a list of 80 concrete nouns in English, with word length varying between 5 and 10 characters. Two separate lists were used, one for each testing session, with order of these lists counterbalanced across subjects. The words were those employed by Van Overschelde and colleagues [56], complemented with help of a dictionary.

Words in each list were presented either in a standard font (Courier New, 60 instances) or a novel font (20 instances). Novel font words had a variable color (one of ten possible colors, with each color repeated twice within the list), variable typeface (unique for each novel word within a list), and a larger size.

Each list was shown twice in each testing session, with no change in order, font, or color, and participants took a short break after the first presentation. The words were presented in the middle of a gray screen (size 21″) located 80 cm in front of the subject, such that standard words (font size 17) subtended 2.5 to 5 degrees of visual angle, depending on word length, and novel words (font size 30) 5.7 to 9.6 degrees of visual angle.

Each trial started with the presentation of a fixation cross for a random interval of 400 to 500 ms (uniform distribution). A word was subsequently presented in the middle of the screen and remained visible for 3500 ms.

In the study phase, participants were instructed to learn the words. In the cued recall phase, participants were provided with cues for 40 of the previously learned words (the 20 novel words and a random 20 of the standard words – not all standard words were cued to reduce the duration of the task). Cues consisted of the first two letters of each word, presented one at a time in random order, and participants completed the studied word by typing the remaining letters. Each of the studied words had a unique combination of first two letters.

In addition to the visual word stimuli, during the study phase an auditory stimulus was presented following the visual onset of each word after an interval. The interval between visual and auditory onset was randomly selected from a uniform distribution of 817 to 1797 ms. Sounds were of two types; either a standard ‘beep’ tone (2.2 KHz, 300 ms), which was presented in 58 out of 80 trials, or a trial-unique sound clip (300 ms), which was presented in 22 out of 80 trials. There was no relationship between the auditory stimuli and the visual words, and participants were instructed to ignore the sounds. The auditory stimuli were included in the experimental design to yield an independent measure of novelty processing, but, consistent with other subsequent results from our lab, there was no evidence in the data of differential processing of standard tones and unique sound clips and this manipulation is not discussed further.

### EEG Recordings and Data Analysis

EEG was recorded from 128 scalp locations using the BioSemi Active2 system (BioSemi, Amsterdam, The Netherlands). Electrodes were placed according to the radial ABC BioSemi montage. Vertical electro-oculogram (EOG) was additionally recorded from 2 electrodes placed 1 cm. lateral to the outer canthi of each eye, horizontal EOG was recorded from 2 electrodes placed above and below the right eye, and reference signals were recorded from electrodes placed over the right and left mastoids. The sampling rate was 512 Hz. The Biosemi is a driven-right-leg amplifier, rather than a traditional differential EEG amplifier, and thus does not employ ground electrodes.

Analysis was performed with EEGlab [57] and custom-written Matlab scripts. EEG data was re-referenced to the average of the signal from the two mastoid electrodes, resampled to 500 Hz, digitally filtered (0.05–40 hz; finite impulse least-square kernel with 6 db transition of 0.01 hz. for low-pass filter and 6 db transition of 2 hz. for high-pass filter), and baselined on the 100 ms interval preceding stimulus onset.

Independent components analysis was computed from epoched data collapsed across conditions [58], [59]. Components accounting for blink artifacts were manually identified and removed from the data, and trials showing substantial muscle artifacts were also identified and rejected from further analysis (threshold for rejection was set to 100/−100 µV). This resulted in the rejection of approximately 5% of data per subject, and subsequent analyses are based on per-subject averages of a.) 37 novel trials in the drug condition, b.) 38 novel trials in the placebo condition, c.) 112 standard trials in the drug condition, and d.) 116 standard trials in the placebo condition.
